# Perspective on Improving the Relevance, Rigor, and Reproducibility of Botanical Clinical Trials: Lessons Learned From Turmeric Trials

**DOI:** 10.3389/fnut.2021.782912

**Published:** 2021-12-03

**Authors:** Janet L. Funk, Claus Schneider

**Affiliations:** ^1^Department of Medicine, University of Arizona, Tucson, AZ, United States; ^2^Department of Pharmacology, Vanderbilt University, Nashville, TN, United States

**Keywords:** botanical, clinical trial, curcumin, turmeric, curcuminoids, dietary supplement

## Abstract

Plant-derived compounds, without doubt, can have significant medicinal effects since many notable drugs in use today, such as morphine or taxol, were first isolated from botanical sources. When an isolated and purified phytochemical is developed as a pharmaceutical, the uniformity and appropriate use of the product are well defined. Less clear are the benefits and best use of plant-based dietary supplements or other formulations since these products, unlike traditional drugs, are chemically complex and variable in composition, even if derived from a single plant source. This perspective will summarize key points–including the premise of ethnobotanical and preclinical evidence, pharmacokinetics, metabolism, and safety–inherent and unique to the study of botanical dietary supplements to be considered when planning or evaluating botanical clinical trials. Market forces and regulatory frameworks also affect clinical trial design since in the United States, for example, botanical dietary supplements cannot be marketed for disease treatment and submission of information on safety or efficacy is not required. Specific challenges are thus readily apparent both for consumers comparing available products for purchase, as well as for commercially sponsored vs. independent researchers planning clinical trials to evaluate medicinal effects of botanicals. Turmeric dietary supplements, a top selling botanical in the United States and focus of over 400 clinical trials to date, will be used throughout to illustrate both the promise and pitfalls associated with the clinical evaluation of botanicals.

## Introduction

Research of plant-derived products (e.g., extracts and/or dried plant parts) stands at the complex intersection of science, consumerism, industry, and federal regulation connecting stakeholders with differing and only partly overlapping interests and expectations. Nowhere is this more apparent than when examining the design and results of published botanical clinical trials and their therapeutic impact (1). In contrast to FDA-approved drugs, the regulatory environment for botanical dietary supplements in the United States (US), which only allows their sale under the explicit provision that the products not be marketed for the treatment or prevention of any specific disease, does not provide a strong commercial incentive for financing appropriately powered and designed (e.g., dose finding or equivalency) clinical studies (2, 3). The often-lacking defined chemical composition of botanical products, as well as their non-uniformity, adds a layer of complexity for scientists, clinicians, and consumers alike when attempting to understand the medical implications of published trials (4). Thus, consumers often become the final arbiters of information derived from trials of readily available botanicals, and may use a product with a chemical composition distinct from that studied to treat a medical condition for which definitive efficacy and safety data are also lacking (5).

While approximately one third of the earth's plants have been used traditionally as medicines, often in combination, <10% of traditional medicinal plants have been the focus of scientific research (6). Despite this absence of scientific evaluation, a majority of populations in developing nations continue to rely on traditional remedies for disease treatment, while in developed nations, such as the United States, over the counter botanical sales continue to expand (7). How can we best marshal limited commercial and government resources to improve the quality and significance of information derived from botanical clinical trials to better understand the benefits and limitations of plant-derived products? Using turmeric as a test case, given its rich history of ethnobotanical use (8), the impressive number (>400) of modern clinical studies conducted to understand its best use (9, 10), and its current rank as one of the top selling botanicals in the United States (4, 11), we will summarize key points to be considered when designing or evaluating results from botanical clinical trials.

## Scientific Premise Supporting Clinical Evaluation of a Botanical

### Ethnobotanical Evidence

In contrast to pharmaceutical development, which usually begins with a specific biological target and works backwards to find a silver bullet, clinical evaluation of botanicals often has its nidus in ethnobotanical evidence of therapeutic effects of a particular plant, mechanism unknown (12). Indeed, the majority of plant-derived compounds developed into pharmaceuticals were identified following ethnobotanical leads (6). For some plants, centuries of use by specific populations, often supported by written texts, provides a compelling source of information for disease-specific treatments despite an absence of modern studies to confirm effects. Turmeric is one such plant, having been used as an anti-inflammatory in Ayurvedic medicine for thousands of years, up until the present (8). Using modern scientific methods, turmeric clinical trials have offered evidence in support of this traditional anti-inflammatory use (9, 13). While likely not common in antiquity, obesity-associated diseases, like insulin resistance or non-alcoholic fatty liver disease, for which inflammation is a key driver, have been the most studied conditions in turmeric clinical trials, representing almost one third of citations and yielding strong evidence of efficacy (13). Anti-inflammatory effects of turmeric are also strongly supported by studies related to musculoskeletal diseases, the second most commonly studied condition, half of which have focused on osteoarthritis, with the majority of studies reporting clinical improvements (13).

Reliance on ethnobotanical evidence can have limitations, however. For clinically silent disease processes, such as age-related bone loss, ethnobotanical footprints do not exist. In these cases, mechanistic pre-clinical studies in the same or mechanistically similar conditions can sometimes provide direction. For example, in the course of conducting pre-clinical turmeric studies documenting remarkable *in vivo* anti-arthritic efficacy, our laboratory identified direct and indirect inhibitory effects of turmeric on the formation of bone resorbing osteoclasts (14), key mediators of bone loss across all disease states (14–16). Subsequent pre-clinical studies by our laboratory verified anti-resorptive effects of turmeric in a model of menopausal bone loss, a clinically silent disorder, that were subsequently confirmed clinically (16, 17). For other biological processes, such as menopause, symptomatology can be culturally dependent (18), and pharmacogenetic differences between populations can also impact botanical responses (19), a caveat that should be kept in mind when designing–and perhaps most importantly–when interpreting clinical trial results. Similarly, for clinical endpoints more responsive to placebo effects, ethnobotanical evidence may also be less reliable. Menopause again provides a possible example (20), as evidenced by the NIH-funded HALT trial testing black cohosh effects on menopausal vasomotor symptoms where a clinically significant 30% reduction in symptoms was documented in black cohosh—and in placebo—trial arms (21). While placebo responsiveness was not necessarily the reason that this trial did not identify an effect (e.g., criticism of the product used and limited power of the study due to inclusion of multiple arms have also been cited as possible explanations), this caveat must again be considered when designing botanical trials, particularly when estimating effect size to appropriately power the clinical trial.

### Pre-clinical Evidence

Even when ethnobotanical evidence of a medicinal effect is strong, botanical clinical trials are vastly improved when mechanistic data are available from appropriately designed pre-clinical studies, particularly those performed *in vivo* (22). In addition to strengthening scientific premise, mechanistic information can also identify biomarkers for inclusion as endpoints, thus improving assessment of pharmacodynamic efficacy and pharmacokinetic sufficiency. For example, pre-clinical data documenting specific, avid binding of turmeric-derived curcumin to amyloid plaques in brains of Alzheimer's Disease (AD) mice has been leveraged, taking advantage of curcumin's natural fluorescence, to image these plaques non-invasively in the retinas of AD mice (23). Subsequently, curcumin has been used successfully to image retinal plaques in aging patients suffering from cognitive decline (24), suggesting a diagnostic tool for a disease where few currently exist. Since curcumin is also reported to reduce amyloid plaques in AD mice (25), an endpoint now accepted, albeit controversially, by the FDA as a measure of AD pharmaceutical clinical efficacy (26), this pre-clinical discovery suggests that curcumin-visualized changes in retinal plaques could serve as a biomarker for clinical trials assessing the efficacy of drugs–including curcumin–in slowing AD progression.

**Figure 1:**
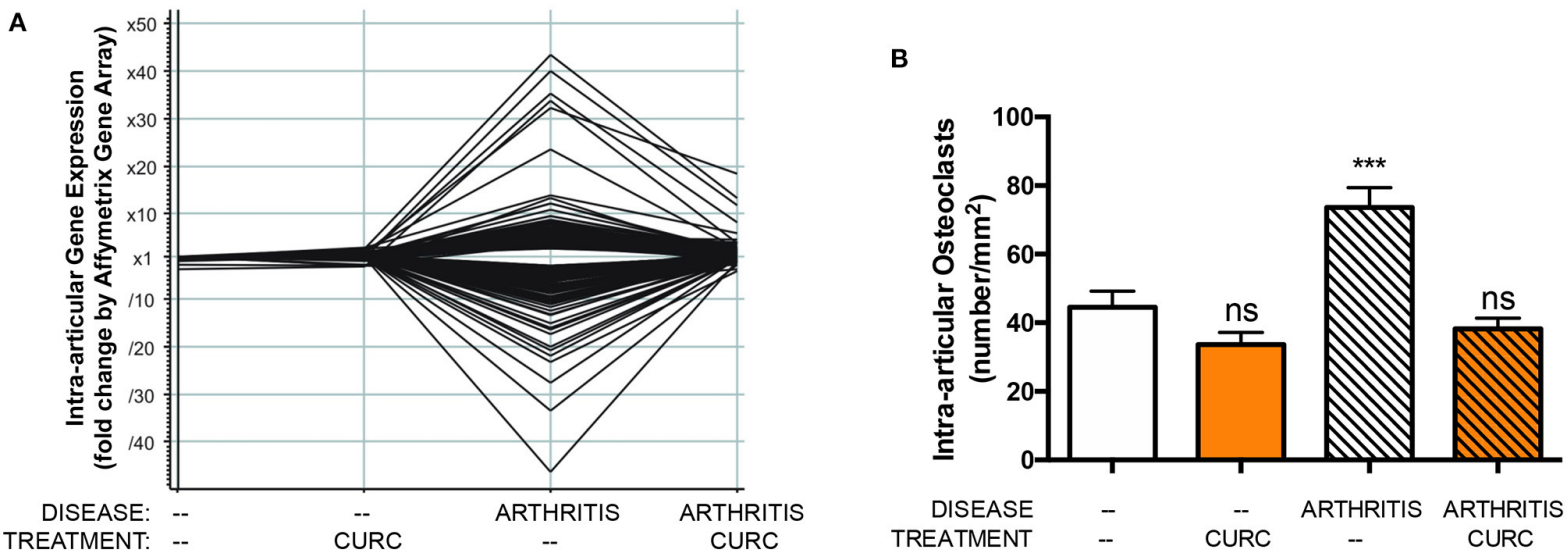
In a pre-clinical arthritis model, consistent with curcuminoid blockade of intraarticular NF-κB activation (not shown), curcuminoid (CURC) treatment significantly altered **(A)** gene expression in arthritic joints, including suppression of over 40 NF-κB regulated genes, and **(B)** inhibited bone-resorbing osteoclast formation in arthritic joints, which is also NF-κB mediated. ns, not significant or ^***^p < 0.001 vs. control (13).

Similarly, *in vivo* and *in vitro* pre-clinical studies from our own laboratories have demonstrated *in vivo* inhibition of NF-κB activation by curcumin (14), an effect likely attributable to adduct formation between oxidative curcumin metabolites and IκB kinase β (IKKß), the activating kinase upstream of NF-κB (14, 27–29). Furthermore, our laboratories have demonstrated that *in vivo* inhibition of NF-κB activation in a pre-clinical arthritis model is associated with decreased NF-κB-induced cytokines or NF-κB-mediated tissue destructive processes (i.e., formation of bone-resorbing osteoclasts) known to be closely linked with adverse clinical outcome (Figure 1) (14–16). Consistent with these pre-clinical findings, in clinical trials assessing curcumin effects on diseases, such as arthritis, where NF-κB activation is known to contribute to pathology, inhibitory effects of curcumin on NF-κB activation and NF-κB downstream pathways have also been reported, with these biomarkers lending credence and mechanistic support to beneficial clinical outcomes (17, 30–33). Indeed, given the central role of NF-κB in mediating inflammation and the significant contribution of inflammation to many disease processes (34), it is perhaps not surprising that benefits of turmeric have been reported in clinical trials across disease types, consistent with its traditional use as an anti-inflammatory (9).

## Choice of Botanical Product for Study

### Botanical Product Composition

One fundamental feature of botanicals not always appreciated by medical researchers is their chemical complexity and variability, even for products derived from the same plant (35). Most plant-derived medicinal compounds are so called secondary metabolites lacking a function within the plant itself, phytoestrogens being one excellent example (36). Secondary metabolites are directed outward (e.g., polyphenolic curcuminoids in turmeric rhizomes), often as a defensive mechanism, protecting the plant from herbivores, insects, or pathogens; thus, their biosynthesis is context-specific and highly regulated but also variable (36, 37). However, even for well-studied plants like turmeric, where curcuminoids have been identified as a primary bioactive principle and are used for extract standardization (4), so called entourage effects are possible (38), with bioactivity resulting from additive and synergistic effects of component parts. In the case of turmeric, ground rhizome—containing polyphenols (3% curcuminoids by weight), terpene-rich essential oils and polysaccharides—is used both in cooking and for preparation of traditional medical formulations (39), whereas the content of most US turmeric dietary supplements is limited to curcuminoids only (98% curcuminoids by weight) (4).

**Figure 2:**
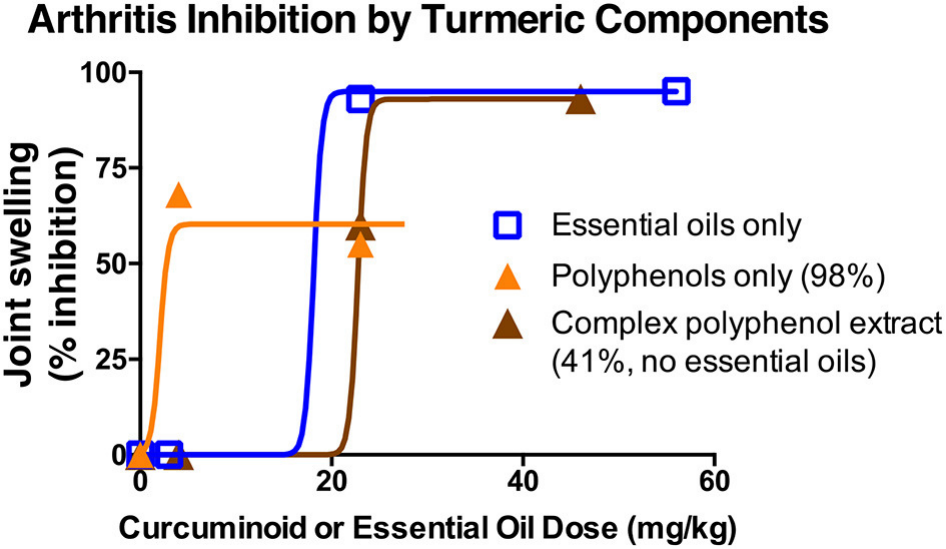
Differential anti-arthritic effects of components extracted from turmeric rhizome in a pre-clinical arthritis model. The anti-arthritic effects of turmeric extracts of different chemical composition were assessed using an ip dosing strategy given reports of altered oral curcuminoid bioavailability when combined with essential oils. Each of turmeric's secondary metabolites [curcuminoids (orange triangles) and essential oils (blue squares)] had significant anti-arthritic effects when administered separately. Interestingly, differential effects were noted for a chemically complex curcuminoid extract (brown triangles) devoid of essential oils but containing polar compounds, such as polysaccharides. Anti-arthritic curcuminoid efficacy was confirmed with oral dosing [50% inhibition; human equivalent dose (HED) of 1 g/d], while protection from oral essential oils was much reduced (20%; HED of 5 g/d) (13).

Given reports of enhanced curcuminoid bioavailability when combined with turmeric's essential oils (40), as well as pre-clinical evidence from our laboratories of enhanced or differential *in vivo* bioactivity of polyphenols derived from turmeric (curcuminoids), or from the botanically-related plant ginger (gingerols), when combined with essential oils and/or polysaccharides, it is readily apparent that botanical extracts, even when standardized to an active principle (e.g., curcuminoids or gingerols) may have differential effects (8, 14, 41–46). For example, in pre-clinical arthritis studies testing turmeric rhizome extracts normalized for curcuminoid or essential oil content (Figure 2), (14, 41, 42, 46) our laboratories have demonstrated anti-arthritic effects for each type of secondary metabolites, as well as additional effects of polar rhizome constituents. However, when testing clinically-relevant, oral doses of purified curcuminoids vs. essential oils, purified curcuminoids were more potent with greater effects (41, 42, 47). In addition, it was notable that *in vivo* anti-inflammatory effects of these same extracts differed for joint vs. hepatic inflammation in the same animals, and also did not necessarily correlate with *in vitro* screening assays (14, 42). Thus, while high throughput screening methods to identify target-specific bioactivity of complex extracts are being developed (48), the utility of pre-clinical data evaluating *in vivo* efficacy of normalized botanical constituents administered alone or in combination can be particularly helpful in choosing a product for clinical study. In addition, entourage effects may also alter active principle bioactivity. The availability of head-to-head pharmacokinetic studies for botanical products normalized to an active principle can serve as a gold standard in this regard. For example, turmeric essential oils, while perhaps of limited anti-inflammatory efficacy in clinically relevant doses, have been variably reported to enhance curcuminoid bioavailability in human pharmacokinetic studies (40, 47, 49). This raises interesting questions not only about botanical product choice for clinical testing, but also regarding assessment of ethnobotanical evidence. If true, the western reductionist approach of using purified curcuminoids rather than complex extracts may not only require higher dosing, but also suggest a corollary question; can intake of lower curcuminoid doses via dietary or traditional medicinal use of essential oil- and curcuminoid-containing turmeric preparations yield biological effects? While this question remains unanswered, it is intriguing that a recent pharmacokinetic study by Mahale et al. (39), examining a turmeric rhizome dose in food analogous to estimated daily dietary intake in India, documented serum curcuminoid levels similar to those reported for therapeutic doses of purified curcuminoid dietary supplement formulated by other means to enhance bioavailability (49–51).

Standardization of the entirety of a plant extract can be difficult, however, because the exact chemical composition can also be dependent not only on the plant and plant part used, but also on growing conditions and method of preparation, including possible fractionation and/or solvents used for extraction, which can differ between products and manufacturers (4, 44, 46). For example, residual levels of 7 different carcinogenic class 1 or toxic class 2 solvents, while below USP limits, were documented by our laboratories in the majority of turmeric dietary supplements tested, suggesting differential modes of preparation, as well as the potential for safety concerns (4). Even when bioactive content is well documented, other aspects of product formulation can confound comparisons of bioactivity in clinical trials and must be considered in clinical trial design. For example, our laboratories have documented that more than half of commercial turmeric dietary supplement sold in the US are enhanced bioavailability formulations and/or include additional botanicals (4). Country-specific regulatory environments can add another layer of complexity to product standardization for botanical clinical trials (3). Reports of botanical product mislabeling in terms of both plant species and chemical content, deliberate adulteration with drugs, or contamination are not uncommon (52). Even in well studied proprietary botanical products, formulations can change over time, possibly altering bioavailability and bioactivity of the standardized active principle. For all of these reasons, besides careful consideration and justification of botanical composition to be tested in a clinical trial, it is absolutely critical that the chemical composition of the specific product and lot(s) used are documented *independently* by the clinical trial researchers *and* reported as an integral part of the clinical trial results—even when commercial products are used—so that research can be replicated and reasons for possible differences between studies can be more rigorously assessed (53). For assay of some active principles, such as curcuminoids (54), standardized methods have been described. In all cases, methodology used to determine product content should be included when reporting clinical trial results.

### Botanical Product Dosing

As exemplified by the turmeric clinical trial literature (9), even for diseases where botanical clinical efficacy is reported across a majority of clinical trials (e.g., curcuminoid treatment of diseases attributable to obesity-associated inflammation or joint-inflammation) (13), definitive conclusions as to efficacy (e.g., from metanalyses) or informed clinical use by consumers are often limited since botanical clinical trials often test only a single dose, with the added complication of disparate products being tested across trials for a given clinical condition. In the case of turmeric dietary supplements, for example, because many are formulated as enhanced bioavailability products (4), curcuminoid dosing is difficult to compare across trials even if product curcuminoid content is reported (9). Thus, neither consumers nor biomedical researchers can easily extrapolate information from a given study to support the rational clinical use or clinical evaluation of a different product, unless detailed information on product composition, dosing and pharmacokinetics are all included in clinical trial design and reported and discussed when publishing results. For example, while osteoarthritis (OA) is one of the most commonly studied diseases in turmeric clinical trials (*n* = 35 unique citations), yielding generally positive effects in studies that are primarily double-blinded, placebo-controlled, and randomized (77%), the OA clinical trials evaluated approximately 20 different, primarily proprietary, curcuminoid-enriched products without any head to head comparisons or inclusion of pharmacokinetic endpoints; rarely included multiple dosing arms; and frequently omitted information regarding curcuminoid content of the study drug and/or rationalization of the single dosing choice (i.e., anticipated bioequivalency of proprietary enhanced bioavailability products, based on prior pharmacokinetic analyses) (13). Thus, both clinical translation and validation of trial results can be improved when dosing information is clearly stated, well justified, and preferably supported by pharmacokinetic data. Both pre-clinical (scaled for human equivalent dosing [HED]) or clinical pharmacokinetic and pharmacodynamic data can be used to optimize clinical trial dosing. For example, a least effective dose of 4 mg/kg daily curcuminoids blocked joint swelling in a rat arthritis model in our laboratory, yielding no greater effect at a higher dose, with a similar inhibitory effect documented with an oral HED of 1 g/d (Figure 2). As even oncologic drugs are sometimes insufficiently studied to determine least effective clinical dose (55), these types of pre-clinical data can help direct botanical clinical trial design.

### Pharmacokinetic Analyses

Inclusion of pharmacokinetic endpoints in clinical trial design can help to overcome limitations attributable to the testing of disparate products across trials, facilitating comparisons. This is most particularly true for botanicals, such as curcuminoids, where use of enhanced bioavailability products is common and careful head to head pharmacokinetic comparisons of formulations containing identical amounts of the bioactive are required to determine actual bioequivalency (50). Even when bioequivalency or bioavailability has been reported previously for a product—and most definitely in cases where it has not—as a minimum standard, rudimentary assessments of bioavailability (e.g., assessment of Cmax, the maximum plasma concentration) should be included in clinical trial design. For example, while different approaches have been used to enhance curcuminoid bioavailability, targeting absorption or secondary metabolism (40, 49–51, 56), a rare head to head comparison of different proprietary enhanced bioavailability curcuminoid products in healthy adults did not support prior published pharmacokinetic reports in all cases (49, 56). This demonstrates the importance of documenting Cmax or other pharmacokinetic parameters in clinical trials, particularly when testing botanical products in disease-specific populations.

**Figure 3:**
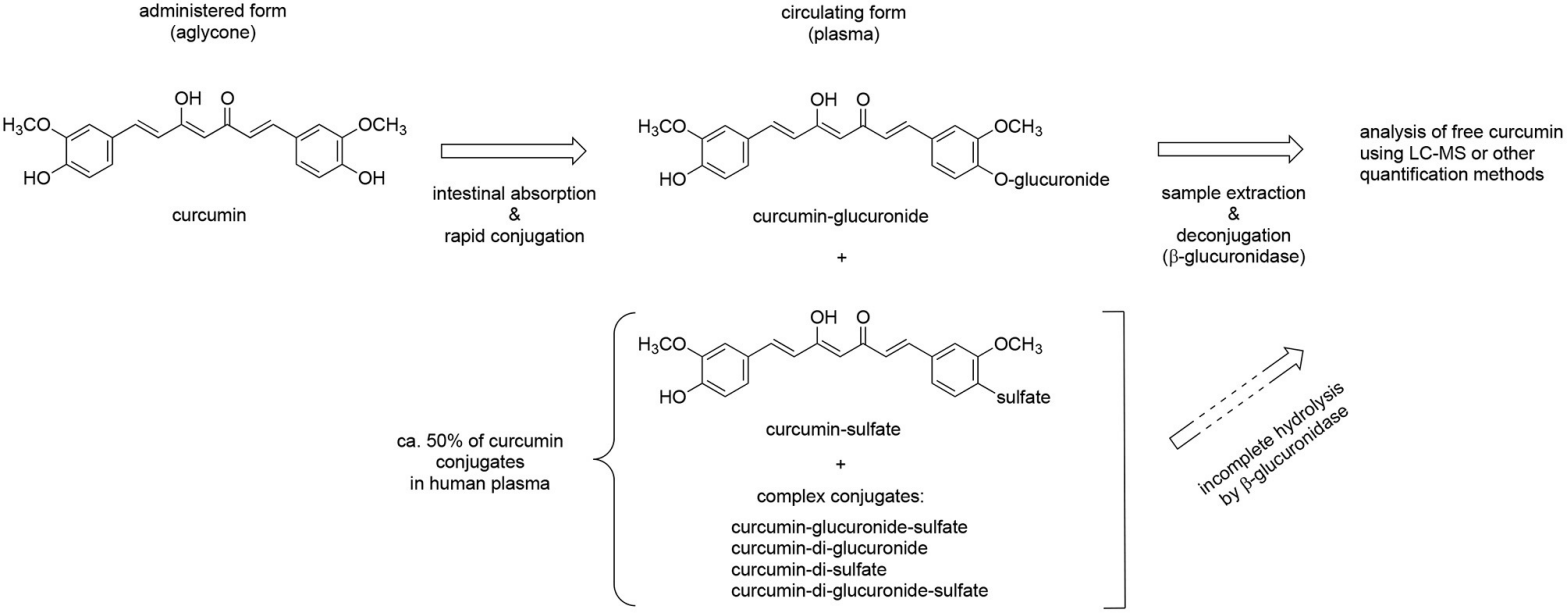
Metabolic conjugation of curcumin and deconjugation for quantitative analysis. Curcumin is consumed in its free (aglycone) form and undergoes rapid phase II conjugation following intestinal absorption. The glucuronide conjugate accounts for about half of the circulating conjugates while sulfate and other, more complex conjugates account for the rest. Free curcumin is low or undetectable in plasma samples. For quantification plasma samples are often deconjugated using β-glucuronidase but this achieves only incomplete hydrolysis of sulfate and complex conjugates; more complete hydrolysis of all conjugates can be achieved using sulfatase. Direct analysis of conjugates is preferred but hampered by the large number of conjugates, lack of standards, and technical challenges. Reduction of the aliphatic double bonds, a significant route of metabolism *in vivo*, and other metabolic events are not illustrated.

**Figure 4:**
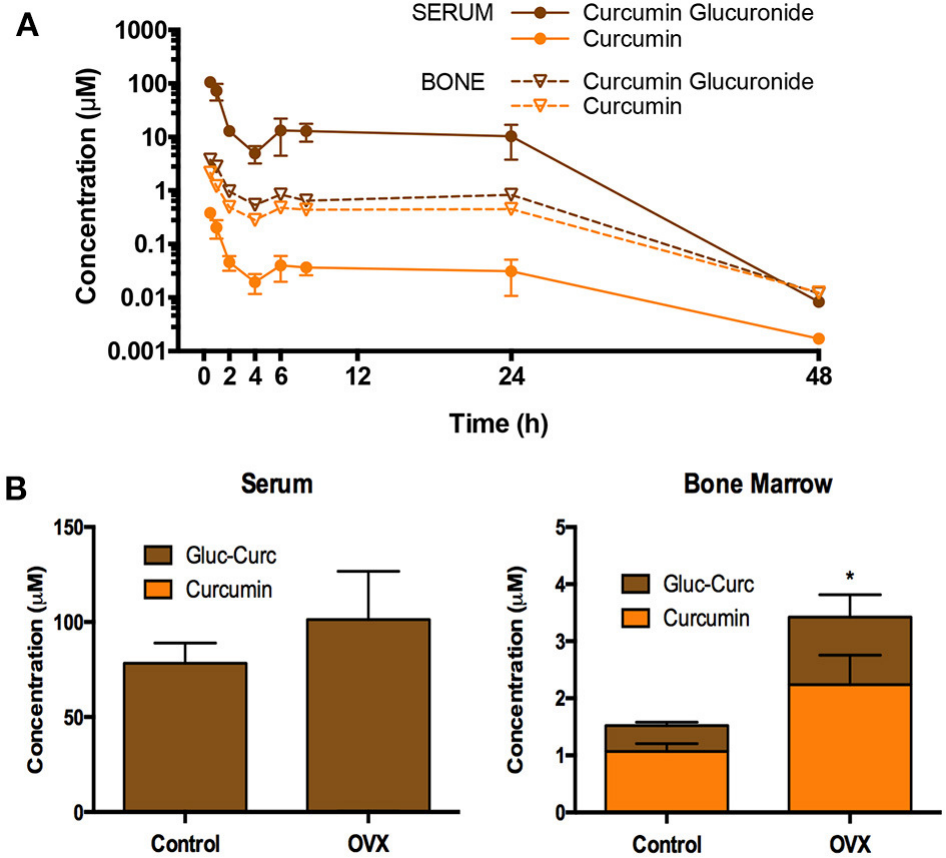
Pharmacokinetics of oral curcuminoids in mice. **(A)** Circulating curcumin-glucuronide (CG) levels predominate and are sustained for up to 24 h in mice following a single oral curcumin dose (HED 2.5 g), with sustained, albeit lower, levels also documented in bone, where ß-glucuronidase-dependent GC hydrolysis to form aglycone curcumin occurs, resulting in higher aglycone concentrations than those perfusing bone. **(B)** Following curcumin ingestion in mature ovariectomized (OVX) mice modeling menopausal bone loss (vs. controls), the capacity of bone to deconjugate curcumin glucuronide distributing to this site persists and is higher in OVX mice. Interestingly, curcumin concentrations in mouse bones are also highest in trabecular bone compartments where menopausal bone loss is most pronounced. Figures are reproduced with permission from John Wiley and Sons (62).

The design of pharmacokinetic endpoints in botanical clinical trials also can present unique challenges since the *in vivo* metabolic fate of plant-derived compounds can complicate analyses. For example, we and others have demonstrated that curcumin and many other plant-derived polyphenols primarily circulate as glucuronide or sulfate conjugates (57–60), with ingested aglycones being near undetectable (Figure 3). Indeed, in the case of curcuminoids, these conjugates can persist in the circulation for over 24 h (e.g., 10% of administered curcuminoids, independent of dose) (61), due in part to enterohepatic recirculation (Figure 4A) (60–65). For this reason, and because glucuronide conjugates are difficult to analyze (66), serum samples for curcuminoids and other botanicals are often pre-treated with deconjugating enzymes to liberate the aglycone prior to pharmacokinetic analyses (51, 58). This practice, however, is not always well documented or characterized, nor are the clinical implications of low *in vivo*, bioactive aglycones always considered (60, 67). For example, data from our laboratories indicate that the common practice of glucuronidase hydrolysis can underestimate curcumin exposure due to incomplete hydrolysis of significant quantities of sulfated or higher order conjugates and suggest the use of sulfatase instead of glucuronidase since the former enzyme achieves a more complete hydrolysis of conjugates (58). Some have questioned the clinical relevance of such measures as conjugates typically lack bioactivity (68). Others postulate that the prolonged circulation of these conjugates provides a ready source of material (e.g., polyphenols) that can be deconjugated locally, and most particularly at sites of inflammation due to the presence of glucuronidase-rich hematopoietic cells, to form the bioactive aglycone (60, 69). Evidence for this later postulate has come from recent studies in our own laboratories. Following oral curcumin administration to mice, bone has the capacity to deconjugate the majority of circulating curcumin glucuronides distributing this site (Figure 4B), which has high levels of glucuronidase due to resident hematopoietic marrow cells (60, 62, 67). This deconjugation process is glucuronidase-dependent and can yield local aglycone curcumin concentrations sufficient to inhibit NF-κB-mediated formation of bone-resorbing osteoclasts (60, 62, 67).

### *In vivo* Botanical Metabolism

**Figure 5:**
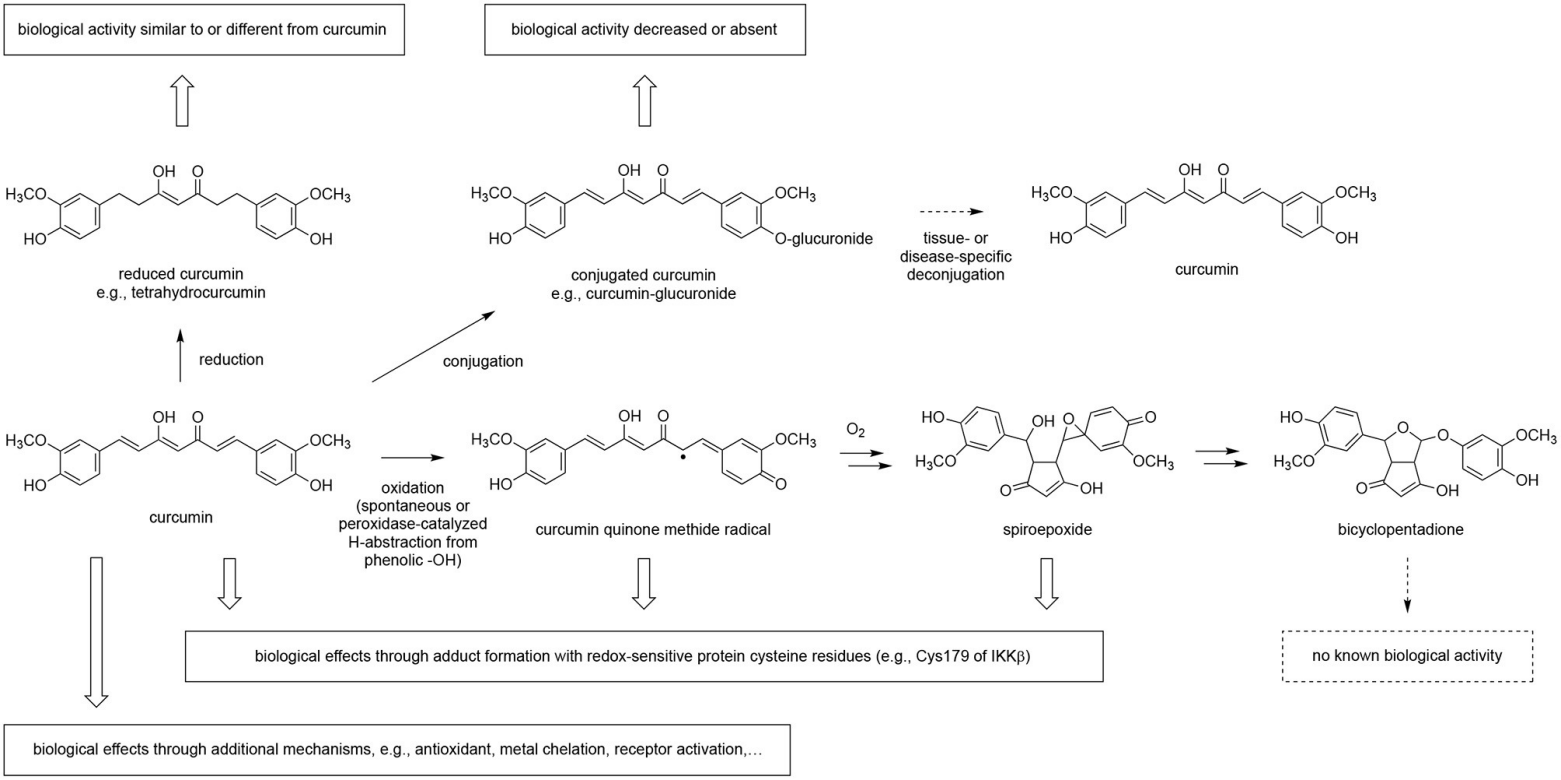
Metabolism of curcumin and the effect on biological activity. Curcumin is reduced and conjugated *in vivo* as shown by the detection of the corresponding metabolites in plasma samples. Deconjugation of circulating and inactive curcumin-glucuronide by β-glucuronidase may contribute to a tissue- or disease-specific effect. Oxidation of curcumin is prominent in buffer and in cultured cells but awaits to be proven *in vivo*. Adduction of curcumin to protein has been described, and at least in part depends on oxidation of curcumin to a quinone methide or other electrophilic oxidation product, i.e., spiroepoxide, that target redox-sensitive cysteine residues and soluble thiols like glutathione. The quinone methide radical and spiroepoxide are unstable intermediates in the oxidation of curcumin to the stable end-product, bicyclopentadione. Curcumin also exerts biological effects through mechanisms not involving metabolic transformation.

A further complication in assessing botanical exposure is the possibility that botanicals, besides forming phase II conjugates, may undergo further *in vivo* metabolism to create additional bioactive moieties (28, 70, 71). This has been extensively described for flavonoids (72–74), and curcumin is also susceptible to reductive as well as enzymatic and non-enzymatic oxidative metabolism (Figure 5) (66, 75). Again, in the case of curcumin, our laboratories have demonstrated an important role for oxidative metabolites of curcumin (70, 76) in altering protein function via the formation of specific adducts (27, 77–80). While evidence for protein adduction of curcumin *in vivo* is yet lacking, in cell-based assays multiple proteins appear to be targeted by reactive oxidative metabolites of curcumin (28, 67, 77–83), consistent with curcumin's reported pleiotropic effects. Protein adduction appears specific and reproducible, likely dictated by the susceptibility of specific proteins (e.g., regulatory site cysteine thiols) to reaction with the existing enone electrophile of curcumin or with electrophilic moieties in metabolites formed upon oxidative transformation (71, 83, 84). For example, curcumin blockade of NF-κB, a transcription factor that is a master regulator of inflammation, appears attributable to adduct formation with the Cys179 residue of IKKβ, the upstream kinase controlling NF-κB activation (85). This tendency to form covalent protein adducts causes some plant-derived compounds, such as curcumin or flavonoids (74, 76), to be “frequent hitters” in screening assays, leading some to suggest that these compounds should be avoided in drug discovery or, indeed, biomedical research (86, 87). However, covalent modification is a pharmacologic strategy employed by many FDA-approved drugs (88, 89), most notably kinase inhibitors (90), or widely used drugs like proton-pump inhibitors (91), anti-thrombotics targeting the platelet P2Y12 receptor like clopidogrel (92), and the cyclooxygenase inhibitor aspirin (93). Thus, it can be argued that the clinical evaluation of botanicals, such as curcuminoids, that specifically, albeit not exclusively, target proteins physiologically relevant to their ethnobotanical use via this mechanism (e.g., blockade of NF-κB via covalent kinase inhibition), can be justified, particularly when the scientific premise is further supported by pre-clinical evidence of *in vivo* efficacy without “off target” toxicities (14, 16).

Given the major effect that *in vivo* metabolism of botanicals can have on bioavailability and bioactivity, this not only complicates the design and interpretation of relevant pharmacokinetic assays, but also raises questions about possible pharmacogenomic differences between subjects in botanical clinical trials that could alter clinical outcomes. For example, in the case of polyphenols, such as curcumin, that rapidly undergo phase II metabolism, genetic variations in endogenous conjugation (e.g., defective conjugation due to UGT1A1 mutations [Gilbert Syndrome], affecting almost 10% of adults) and/or deconjugation capacity may be important determinants of botanical bioavailability, and thus bioactivity, which should be considered in clinical trial design (60, 62, 94–96). Interestingly, separate reports suggest that gender may also be an important determinant of clinical curcumin responses, independent of bioavailability, and that gender may also influence bioavailability, although differences in body weight may have accounted for higher levels documented in women. This finding remains clinically relevant since curcuminoids in clinical trials–and clinical use–are rarely dosed based on weight (50, 97, 98). In addition, for certain botanicals, most notably phytoestrogens (99), but also possibly curcuminoids (100), metabolism by the gut microbiome can also affect bioavailability.

## Other Critical Elements and Potential Barriers to High Quality Botanical Clinical Trial Design

Design elements driving the quality of any clinical trial are obviously also applicable here, including appropriately powered, controlled, randomized and double-blinded studies with pre-specified analyses (101). However, often these elements are overlooked in botanical clinical trials, or, indeed difficult to achieve, whether due to funding limitations, or other issues specific to a botanical. For example, it can be difficult to blind studies, as is the case with curcumin, due to its unique vibrant orange hue. Placebo composition is therefore an important element of botanical study design (21). In our own experience, optimization of placebo composition, particularly when the botanical product is being obtained from a nutraceutical company with fixed production lines, can be a time-consuming issue that should be considered in planning timelines. Another element to be considered in the US, even when testing an over-the-counter product, is the need to prepare, file and undergo an FDA review of an Investigational New Drug application (IND), following botanical specific guidelines (102), if disease outcomes (i.e., disease treatment) are an endpoint, as well as consideration of whether clinical trial goals could be met with an alternative design (103).

Funding is more limited for botanicals than for pharmaceuticals, given their different marketing and approval pathways. This often places limits on study size and duration that impact clinical and statistical significance. For example, the two published trials assessing curcumin in AD which were only of 6-month duration and involved fewer than 30 treated subjects, perhaps not surprisingly, yielded no significant effects, an outcome attributed in part to low product bioavailability (104, 105). In contrast, larger and longer (e.g., 12-month) studies examining effects of enhanced bioavailability curcumin products on cognitive decline in aged adults have all reported benefits (106–111). In the US, industry-funded clinical trials are disincentivized in general, since nutraceuticals can be sold without evidence of efficacy and cannot be marketed for disease treatment (3). Other market driven forces can impact industry-supported study design in ways that are sometimes not helpful to consumers or researchers. For example anti-arthritic benefits have been reported in separate trials for two turmeric products manufactured by the same company, a unique product combining curcuminoids with turmeric polysaccharides and a curcuminoid-only product analogous to most commercial turmeric supplements, without an assessment of bioactivity attributable—or not—to polysaccharide content (112, 113).

## Prioritizing Public Health and Safety

Due to the large number of traditional medicinal plants, the disparate composition of commercial products for a given plant, and the paucity of botanical clinical trial funding, the task of documenting medicinal benefits of every potentially valuable botanical is daunting and likely not achievable. How can available resources best be used? As previously discussed, strong ethnobotanical and pre-clinical evidence of botanical efficacy are important pillars supporting clinical trial design. The study of lesser-known plants, particularly, for diseases lacking effective treatments, can also yield clear benefits. However, in these cases, considering all the intricacies associated with the study of plant-based medicines as described here, strong pre-clinical pharmacokinetic and pharmacodynamic evidence should first be obtained to guide the appropriate design of subsequent botanical clinical trials.

Prevalence of use is one additional factor to consider; public health benefits can be greater in these cases, not only with respect to efficacy, but also safety. Indeed, because some populations tend to use botanicals for disease treatment even in the absence of cultural traditions or evidence of efficacy, examination of safety becomes a key concern. For example, in our recent observational studies, current turmeric use was reported by one third of individuals with rheumatoid arthritis or breast cancer in the US despite a paucity of efficacy or safety data (11, 114, 115). Botanical safety information is thus important for public health. Consumers tend to falsely equate natural with safe, and, in the US, may also incorrectly assume that the federal government requires commercial botanical products to be vetted for efficacy and safety (116, 117). Examination of possible pharmacogenetic risk factors related to botanical metabolism and/or adverse drug-botanical interactions can therefore be important elements of botanical clinical trial design, particularly for government funded studies (118, 119). This is particularly true when studying populations at higher risk of adverse effects due to underlying chronic disease and/or concurrent use of pharmaceuticals, as, for example, has been reported for concurrent use of certain dietary supplements with breast cancer chemotherapy (11, 120, 121).

One additional safety related concern, unique to botanicals (vs. pharmaceuticals) and attributable to their variable content and lack of regulatory oversight, is the risk of adverse effects due to possible contaminants (2, 4). For example, isolated case reports from our laboratories and others of turmeric- or black cohosh-associated hepatitis highlight potential risks, as well as difficulties in determining the etiology, of adverse botanical effects outside the context of clinical trials (2, 122–124). Thus, consideration of all available product- or plant-specific safety data must guide product selection in order to optimize botanical clinical trial design (4, 122). At the same time, well-designed clinical trials are often the only source of high-quality safety information for a given botanical product. For example, a review of FDA MedWatch reports for turmeric obtained by our laboratories under a Freedom of Information Request in 2017 yielded 107 reports, with turmeric products being equally listed as the possible suspected product (being used in combination with other supplements in half of these cases) vs. concurrent medication, making identification of turmeric product-specific safety issues difficult. Lastly, for publicly funded studies, selection of a product representative of those readily available to consumers may also be a consideration (4).

## Conclusion

Plants are a rich source of potential therapeutics, whether developed as drugs, or used as complex botanical products. However, the chemical complexity and differential regulation of botanicals provide unique challenges when designing high quality botanical clinical trials, with perhaps the largest public health and medical benefits to be gained by prioritizing the study of botanicals with a high prevalence of use and/or likelihood of ameliorating diseases lacking effective treatments. Turmeric is one such example, being a top selling botanical already in widespread use with demonstrated promise in the treatment of inflammatory conditions associated with obesity, a major health problem worldwide. However, for many published turmeric clinical trials, key clinical study design elements unique to botanicals, as described here, have been lacking. Thus, while turmeric may appear to be overstudied as compared to other botanicals, because of its widespread prevalence of use and the strength of existing ethnobotanical and scientific evidence of medicinal effects, it can perhaps be best described as *ineffectively* studied from the viewpoint of consumers and healthcare providers. Improved botanical clinical trial designs, making the best use of limited resources, are needed to realize the full potential of turmeric and other medicinal botanicals, complementing the experimental evidence of our ancestors with the application of current best clinical research practices.

## Author Contributions

All authors listed have made a substantial, direct, and intellectual contribution to the work and approved it for publication.

## Funding

Work on botanicals including curcumin in the authors' laboratories has been funded by the National Center for Complementary and Integrative Health (NCCIH) and the Office of Dietary Supplements (ODS) (R01AT006896, R34AT007837, R21AT003614, R21AT004182, R21AT005145, P50AT000474, F31AT004875, F31AT007287, and F31AT009938), the National Cancer Institute (R01CA174926 and R03CA159382), and the National Institute of Arthritis and Musculoskeletal and Skin Diseases (R21AR078424) at the National Institutes of Health (NIH).

## Conflict of Interest

The authors declare that the research was conducted in the absence of any commercial or financial relationships that could be construed as a potential conflict of interest.

## Publisher's Note

All claims expressed in this article are solely those of the authors and do not necessarily represent those of their affiliated organizations, or those of the publisher, the editors and the reviewers. Any product that may be evaluated in this article, or claim that may be made by its manufacturer, is not guaranteed or endorsed by the publisher.
